# Effect of Soy Isoflavone on Prostate Cancer Cell Apoptosis Through Inhibition of STAT3, ERK, and AKT

**DOI:** 10.3390/cimb46110743

**Published:** 2024-11-06

**Authors:** Yoon-Jin Lee, Changyeol Lee, Dongsic Choi, Yeji Lee, Sang-Han Lee

**Affiliations:** Department of Biochemistry, College of Medicine, Soonchunhyang University, Cheonan 31511, Republic of Korea; jefferysun4@naver.com (C.L.); dongsic@sch.ac.kr (D.C.); yjyjlee37@naver.com (Y.L.); m1037624@sch.ac.kr (S.-H.L.)

**Keywords:** genistein, prostate cancer, apoptosis, reactive oxygen species, STAT3

## Abstract

Genistein, an isoflavone found in soybeans, exhibits antioxidant, anti-inflammatory, and anticancer properties. This study explored the molecular mechanisms behind genistein’s anticancer effects in prostate cancer DU145 cells. In this study, genistein decreased cell viability, increased annexin V-PE(+) cells, and enhanced the sub-G_0_/G_1_ peak by flow cytometric analysis. Increased reactive oxygen species increased mitochondrial depolarization indicating mitochondrial dysfunction and inhibition of ATP formation were also observed in genistein-treated DU145 cells. Genistein upregulated p53 at the mRNA and protein levels and increased caspase-3/7 activity along with the cleavage of Bax, procaspase-3, and PARP. With the increasing genistein concentrations, the percentage of cells in the sub-G_0_/G_1_ peak and G_2_/M phase increased, which was inhibited by treatment with the pan-caspase inhibitor Z-VAD together with 100 μM genistein, which had little toxicity to normal prostate epithelial HPrEC cells. Genistein treatment simultaneously inhibited the activation of STAT3 and other closely related oncogenic kinases such as AKT and ERK and p38 and decreased VEGF expression. Taken together, these results suggest that genistein inhibits the growth of DU145 cells and induces apoptosis by inhibiting STAT3, AKT, ERK, and p38 which provides a molecular basis for the anticancer activity of genistein and suggests its potential as a valuable therapeutic candidate for prostate cancer.

## 1. Introduction

The incidence of prostate cancer, the second leading cause of death due to cancer in men, is significantly higher in the United States and Europe than in Asia [1]. More than 95 percent of these cases are adenocarcinomas, which primarily originate in the glandular ducts and very rarely in the ductal regions [2]. Diagnostic methods for prostate cancer include prostate biopsy and analysis, prostate-specific antigen (PSA) testing, rectal examination, and magnetic resonance imaging. Treatment options vary based on the patient’s disease grade and stage, likelihood of recurrence, tumor nature, and PSA levels, and can include chemotherapy, radiotherapy, hormone therapy, surgery, and cryotherapy [3]. Nonetheless, current treatments often result in severe side effects and toxicities, such as low counts of white and red blood cells, hair loss, peripheral neuropathy, erectile dysfunction, and sexual dysfunction, highlighting the need for novel treatment approaches [4,5]. Three cell lines commonly utilized in prostate cancer research are DU145, PC3, and LNCaP [6]. Among these, the DU145 cell line originates from central nervous system metastases of primary prostate cancer and exhibits lower metastatic potential than the PC3 line, characterized by its hormone insensitivity and lack of PSA expression [7].

Recent years have seen an escalating interest in the role of natural compounds as enhancers of chemotherapy, aimed at increasing treatment efficacy and reducing side effects. Phytochemicals, bioactive plant chemicals, are explored for treating various diseases, including cancer [8]. Biologically active compounds in natural products derived from plants, aquatic organisms, and microbes have become major resources for the development of cancer chemopreventive or chemotherapeutic agents by effectively inhibiting cell proliferation, regulating the cell cycle, and interfering with various oncogenic signaling pathways involving phosphoinositol-3 kinases/protein kinase B (PI3K/AKT), mitogen-activated protein kinase/extracellular signal-regulated protein kinase (MAPK/ERK), Wingless and Int-1 (Wnt)/β-catenin, and matrix metalloproteases [A]. Furthermore, natural products can stimulate DNA repair mechanisms through the actions of p21 and/or p53 gene products, including Bcl-2-associated X (Bax) protein, activate caspase cascades, and modulate the synthesis and activity of enzymes or proteins involved in reactive oxygen species (ROS) scavenging and immune surveillance [9,10]. The chemopreventive effects of these natural products may help manage and treat tumors more effectively and may also help reduce the risk of metastasis and recurrence. Among natural products, luteolin, quercetin, apigenin, curcumin, resveratrol, silivinin, epigalocatekin-3-galate, tocotrienol, sulforaphan, ginsenoside, honokiol, and tannic acid have shown excellent efficacy against prostate cancer in vitro and in vivo models [11]. Therefore, scientific interest in discovering effective anticancer agents from natural products is steadily increasing.

Genistein (4′,5,7-trihydroxyisoflavone) exhibits a broad spectrum of pharmacological properties as an isoflavone phytoestrogen in animal cells and acts as a biosynthetic precursor for antimicrobial phytoalexins and phytoantioxidants in legumes. Furthermore, it displays significant anticancer and antioxidant properties, regulating numerous signal transduction pathways at both transcriptional and translational levels [12,13]. Preclinical studies have demonstrated that genistein possesses diverse biological effects such as antioxidant, anti-inflammatory, antibacterial and antiviral activities, influence on angiogenesis and estrogenic effects, and pharmacological impacts on diabetes and lipid metabolism [13,14]. Notably, genistein exhibits anticancer activity against various cancer types, especially breast and prostate cancer [15]. Additionally, it induces apoptosis by increasing caspase-3 gene expression, leading to heightened intracellular protein levels and enzyme activity, potentially minimizing the side effects of chemotherapy and addressing resistance issues associated with continuous drug use [12].

Apoptosis is the process by which a cell ceases growth and division, its contents are not released into the surrounding environment, and ultimately, the cell undergoes controlled death. There are two types of apoptosis: extrinsic pathways that occur through membrane receptor-mediated interactions and intrinsic pathways that begin in the mitochondria by acting directly on intracellular targets [16]. Both pathways involve the p53 protein. Known as a tumor suppressor gene, p53 is inactivated in over half of human cancers. When overexpressed, p53 inhibits the growth of prostate cancer cells and enhances their chemosensitivity; conversely, when p53 is reduced or absent, it may promote the growth of these cancer cells. These tumor suppressive functions are carried out by p53, which is regulated through multiple molecular mechanisms such as senescence, cell cycle arrest, apoptosis, DNA repair, and cellular metabolism [17,18]. Additionally, p53, which is activated in response to DNA damage, UV light, reactive oxygen species, and cytotoxic drugs, blocks cell cycle progression and induces apoptosis, thereby halting uncontrolled cell growth [19,20].

Furthermore, signal transducer and activator of transcription 3 (STAT3) signaling is critical in regulating the cell cycle and apoptosis in cancer. It has been abnormally overexpressed in various cancers, including prostate cancer [21,22]. STAT3 interacts with products such as IL-6 receptors and epidermal growth factor receptor, expressed via a receptor, and promotes growth differentiation of tumor cells while inhibiting tumor cell death. Therefore, targeting STAT3 could effectively suppress cancer [23,24].

Current treatments for prostate cancer in clinical settings face various difficulties. This study was designed to investigate whether genistein induces apoptosis in prostate cancer DU145 cells by activating caspase-3 and enhancing the expression of the tumor suppressor p53. This experiment examined the cell cycle, ROS increase, apoptosis, and p53 expression responses to genistein in prostate cancer DU145 cells compared to normal prostate epithelial HPrEC cells.

## 2. Materials and Methods

### 2.1. Cell Culture

The human prostate epithelial cell line HPrEC and the human prostate cancer cell line DU145 were sourced from the American Type Culture Collection (ATCC; Manassas, VA, USA). HPrEC cells were cultured in prostate epithelial cell basal medium with supplements from the prostate epithelial cell growth kit (ATCC). DU145 cells were maintained in DMEM (Welgene Inc.; Gyeongsan, Republic of Korea) supplemented with 5% fetal bovine serum (FBS). Cells were incubated at 37 °C in a 5% CO_2_ environment until confluence was reached, then the medium was removed, and cells were washed with 1× phosphate-buffered saline (PBS). A 0.25% trypsin-EDTA solution was subsequently added to detach the cells from the culture flask and to transfer them into new culture plates as fresh subcultures.

### 2.2. Cell Viability Assay

Cells were seeded in 96-well plates at 1 × 10^4^ cells/mL in 100 μL of complete medium 24 h before treatment with 0.1% dimethylsulfoxide (vehicle), genistein and/or Z-VAD. 3-(4,5-dimethylthiazole-2-yl)-2,5-diphenyltetrazolium bromide (Sigma-Aldrich Corp., St. Louis, MO, USA) was added to the culture medium, and incubated at 37 °C for 3 h. Absorbance was measured at 540 nm using a GloMax-Multi Microplate Multimode Reader (Promega Corporation, Madison, WI, USA). Cell viability (%) was assessed by comparing with vehicle-treated cells, defined as 100%.

### 2.3. DAPI Staining

The nuclear morphology of apoptotic cells, indicated by nuclear fragmentation and chromosomal condensation, was assessed in cells stained with 2′,7′-dichlorodihydrofluorescein iodide (DAPI, 2 µg/mL) for 30 min in the dark.

### 2.4. Measurement of Intracellular ATP Content

The intracellular ATP content was determined by luminescence using the CellTiter-Glo Luminescent Cell Viability Assay Kit according to the manufacturer’s instructions (Promega Corporation).

### 2.5. Wound Healing Assay

A wound healing assay was conducted to investigate the impact of genistein on cell migration efficacy. When cells achieved 90% confluence in a 6-well plate, a wound track was created in each well, and the cells were rinsed with 1× PBS to remove debris. Subsequently, cells were incubated with 0, 50, 75, or 100 μM genistein for 48 h, and wound closure was monitored under a microscope. Photographs were taken and the width of the wound was measured.

### 2.6. Cell Cycle Analysis

Cell cycle analysis was performed by quantifying DNA content in cells stained with propidium iodide (PI). Cells were trypsinized, centrifuged at 500× *g* for 7 min at 4 °C, and fixed overnight at −20 °C with 70% ethanol. After fixation, cells were washed with 1× PBS, and Muse cell cycle reagent (cat. no. MCH100106; Merck KGaA, Darmstadt, Germany) containing PI and RNAse was added, followed by incubation for 30 min. Data from 10,000 cells were analyzed using a MACSquant analyzer and quantification was performed with MACSQ software version 2.5 (Miltenyi Biotech GmbH, Bergisch Gladbach, Germany).

### 2.7. Annexin V-PE Binding Assay

Apoptotic and necrotic cell distributions were determined using the Muse^TM^ Annexin V & Dead Cell Assay kit (Merck KGaA, Darmstadt, Germany). Cells were harvested, resuspended in the culture medium, mixed with Muse^TM^ Annexin V & Dead Cell reagent, and analysis was performed using a Muse^TM^ Cell Analyzer (Merck Milipore, Billerica, MA, USA).

### 2.8. Caspase-3/7 Activity

Caspase-3/7 activation was quantified using the ApoToxGlo™ Triplex Assay kit according to the manufacturer’s protocol (Promega). Cells were incubated with the caspase-Glo 3/7 assay buffer for 30 min, and luminescence was then measured using a GloMax-Multi Microplate Multimode Reader (Promega, Madison, WI, USA).

### 2.9. Reverse Transcription-Polymerase Chain Reaction (RT-PCR)

Total RNA (1 µg) was converted to cDNA using oligo(dT)_15_ primer and AMV reverse transcriptase (iNtRON Biotechnology, Seongnam, Republic of Korea). The resulting single-stranded cDNA was diluted and subjected to PCR amplification using iMAX IITM DNA polymerase (iNtRON Biotechnology). The PCR was performed using total RNA as the template. The following primers were used to amplify gene fragments. The specific primers for RT-PCR amplification of various genes were as follows: (a) p53 (175-bp): sense 5-ctgccctcaacaagatgttttg-3 and antisense 5-ctatctgagcagcgctcatgg-3, (b) p21 (174-bp): sense 5-atgaaattcaccccctttcc-3 and antisense 5-ccctaggctgtgctcacttc-3, (c) GAPDH (247-bp): sense 5-acctgacctgccgtctagaa-3 and antisense 5-tccaccaccctgttgctgta-3.

### 2.10. Measurement of Reactive Oxygen Species and Mitochondrial Membrane Potential

Cells were seeded in 6-well plates at 10^5^ cells/mL 24 h prior to treating with genistein (0, 50, 75, and 100 μM) in complete DMEM for 48 h. Afterward, cells were harvested by centrifugation at 500× *g* for 7 min and stained with 10 µM 2′,7′-dichlorodihydrofluorescein diacetate (DCF-DA; Sigma-Aldrich) or 30 nM Rhodamine 123 (Sigma-Aldrich) to measure the levels of ROS or mitochondrial membrane potential, respectively, in the dark at 37 °C for 30 min. Subsequently, cells were washed twice with 1× PBS and the average fluorescence intensity of 10,000 cells was measured using a MACSQuant Analyzer (Miltenyi Biotec GmbH).

### 2.11. Western Blot Analysis

Western blot analysis was conducted using cell lysates as previously described [25]. Cell lysates with 40 g of protein were resolved on NuPAGE 4–12% bis-tris polyacrylamide gels (Invitrogen, Carlsbad, CA, USA) and subsequently transferred electrophoretically to Immuno-Blot PVDF membranes. These membranes were incubated overnight at 4 °C with various primary antibodies. After three 15 min washes with Tween-20 in PBS (PBST), the membranes underwent a 1 h room temperature incubation with secondary antibodies conjugated to horseradish peroxidase (HRP) for protein detection. Visualization of the signals was achieved using an ECL detection kit (Cyanagen Srl, Bologna, Italy) and X-ray film. The membrane was re-probed with anti-β-actin (cat. no. A2228, Sigma-Aldrich) antibody to serve as the loading control. Antibodies against p-AKT (cat. no. 9271), AKT (cat. no. 9272), p-ERK (cat. no. 9101), ERK (cat. no. 9102), p-p38 MAPK (cat. no. 9211), p38 MAPK (cat. no. 8690), Bax (cat. no. 5023), B-cell Lymphoma 2 (Bcl-2; cat. no. 2820), poly (ADP-ribose) polymerase (PARP; cat. no. 9542), cleaved PARP (cat. no. 9541), procaspase-3 (cat. no. 9665), and cleaved caspase-3 (cat. no. 9664) were obtained from Cell Signaling Technology, Inc. (Danvers, CO, USA). p53 (cat. no. sc-126), p21 (cat. no. sc-6246), p-STAT3 (cat. no. sc-8059), STAT3 (cat. no. sc-482), vascular endothelial growth factor (VEGF), goat anti-rabbit IgG-HRP (cat. no. sc-2004), and goat anti-mouse IgG-HRP (cat. no. sc-2005) were acquired from Santa-Cruz Biotechnology (Dallas, TX, USA).

### 2.12. Statistical Analysis

Statistical analysis of the experimental data was conducted using one-way ANOVA and Tukey’s post-mortem correction, utilizing SPSS version 17.0 software (SPSS Inc., Chicago, IL, USA). Data are expressed as mean ± standard deviation (S.D.) for the experiment. Differences were deemed significant at a value of *p* < 0.05.

## 3. Results

### 3.1. Effects of Genistein on Cell Viability, Cell Morphology, Nuclear Morphology, and Cell Migration in Prostate Cancer Cells

**Figure 1:**
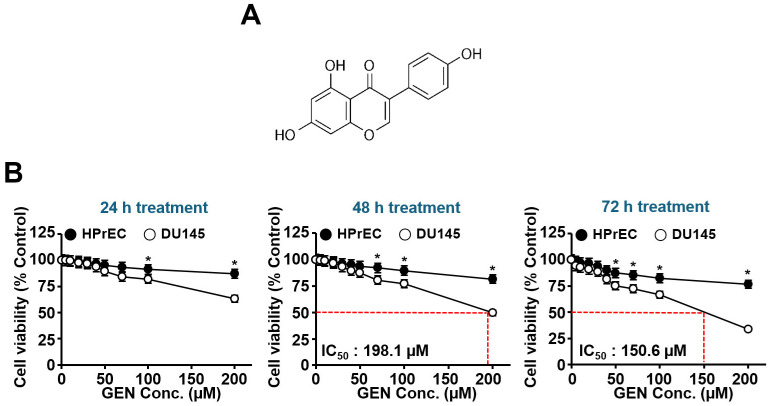

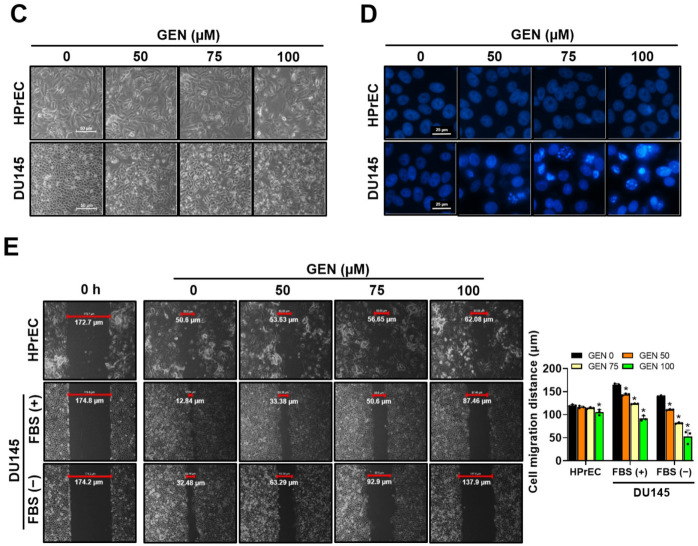
Effects of genistein on cell viability, cell morphology, and nuclear morphology. (**A**) Chem ical structure of genistein. (**B**) Cell viability was measured using an MTT assay in normal HPrEC and DU145 cells. Cells were exposed to increasing concentrations of genistein (0, 5, 10, 20, 30, 40, 50, 75, 100, and 200 μM). (**C**) Cell morphology was assessed under phase-contrast microscopy in HPrEC and DU145 cells. (**D**) Nuclear morphology was evaluated with a fluorescence microscope following DAPI (2 μg/mL) staining in HPrEC and DU145 cells. (**E**) A wound healing assay was conducted. * *p* < 0.05 was compared to respective controls. GEN: genistein, HPrEC: human prostate epithelial cell line, DAPI: 4′,6-diamidino-2-phenylindole, and Conc.: concentration.

To determine the effective doses for genistein (Figure 1A), normal prostate epithelial cell line HPrEC and prostate carcinoma cell line DU145 cells were treated with genistein for 24–72 h, and MTT assay was performed. As depicted in Figure 1B, DU145 cell viability decreased with increasing concentration of genistein; the IC_50_ was 198.1 μM after 48 h of exposure and 150.6 μM after 72 h of exposure. When exposed to genistein at concentrations up to 100 μM for 48 h, cell viability reached over 75%, indicating moderate toxicity in DU145 cells, whereas it showed little toxicity in HPrEC cells. Based on these results, genistein at concentrations of 0–100 μM was used in further studies. After a 48 h treatment with genistein (0–100 μM), significant changes in the morphology of DU145 cells were observed, with a decrease in cell confluency (Figure 1C). An investigation into whether these effects were associated with DU145 cell death included nuclear morphology analysis using DAPI staining (Figure 1D). Treatment with genistein for 48 h showed increased chromatin condensation and nuclear fragmentation in DU145 cells, occurring in a concentration-dependent manner. Subsequent experiments were conducted using genistein up to 100 μM, which caused little toxicity in normal HPrEC cells. The effect of genistein on cell migration and proliferation was examined through wound healing assays on HPrEC and DU145 cells. In this study, genistein inhibited wound filling capacity in a dose-dependent manner (Figure 1E). However, the degree of genistein-induced reduction in wound filling capacity was greater in culture medium that did not contain 5% FBS than in culture medium that contained 5% FBS.

### 3.2. Genistein-Induced Apoptosis Is Mediated by Caspase Activity

**Figure 2:**
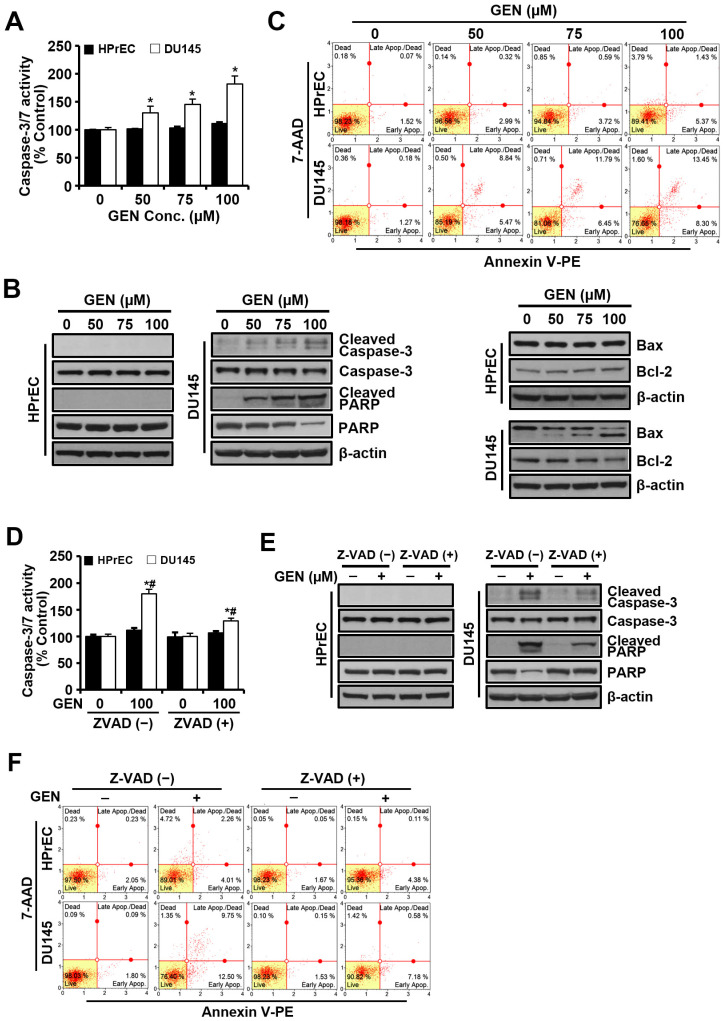
Caspase-3 activation by genistein in HPrEC and DU145 cells. (**A**–**C**) Cells were treated with increasing concentrations (0–100 μM) of genistein for 48 h. Caspase-3/7 activity was determined using an ApoTox-Glo^TM^ Triplex Assay (**A**). Cell lysates were subsequently analyzed by Western blotting with antibodies against procaspase-3, cleaved caspase-3, PARP, Bax, Bcl-2, and β-actin (**B**). The percentage of apoptotic cells after annexin V-PE binding was quantified by a Muse cell analyzer (**C**). (**D**–**F**) Cells underwent treatment with genistein (100 μM) for 48 h in the presence or absence of Z-VAD (10 μM). Caspase-3/7 activity was determined using the ApoTox-Glo^TM^ Triplex Assay (**D**). Cell lysates were then analyzed with Western blotting for procaspase-3, cleaved caspase-3, PARP, and β-actin (**E**). The percentage of apoptotic cells after annexin V-PE binding was determined using a Muse cell analyzer (**F**). Error bars represent mean ± S.D. from three independent experiments. * *p* < 0.05 was compared to respective controls. # *p* < 0.05 was compared to Z-VAD (−) groups. GEN: genistein, HPrEC: human prostate epithelial cell line, and Conc.: concentration.

**Figure 3:**
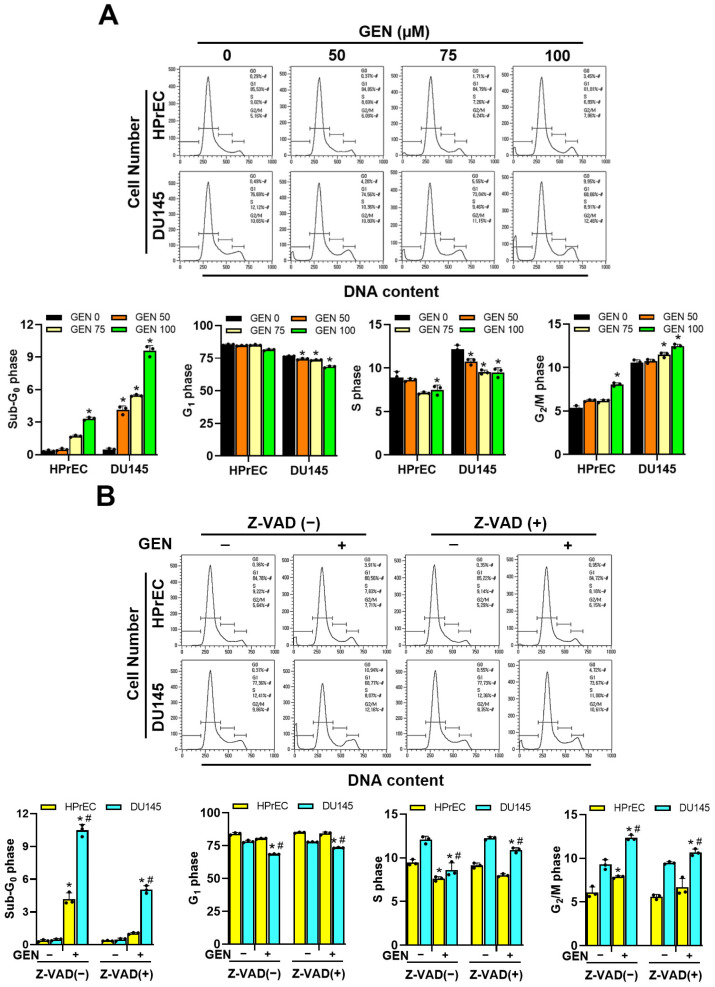
Effects of genistein and pan-caspase inhibitor on cell distribution at sub-G_0_/G_1_, G_1_, S, and G_2_/M phases in HPrEC and DU145 cells. Cells were treated with increasing concentrations (0–100 μM) of genistein for 48 h. Cells were treated with Z-VAD (10 μM) for 2 h prior to incubation with genistein (100 μM) for 48 h. (**A**,**B**) Subsequently, cells were analyzed using flow cytometry after staining with propidium iodide (20 μg/mL). Representative results were sourced from one of three independent experiments. The quantitative data are presented as mean ± S.D. * *p* < 0.05 was compared to respective controls. # *p* < 0.05 was compared to Z-VAD (−) groups. GEN: genistein, and HPrEC: human prostate epithelial cell line.

Caspase-3, a critical downstream effector of the classical apoptosis pathway, is marked by its cleavage during apoptotic cell death activation. To further determine the role of caspases in genistein-mediated apoptosis in DU145 cells, caspase-3/7 activity was measured in cell cytosolic extracts. Genistein treatment resulted in a concentration-dependent increase in caspase-3/7 activity (Figure 2A) and enhanced cleavage of Bax, procaspase-3, and its substrate PARP (Figure 2B). As the concentration of genistein increased, the percentage of cells undergoing the early and late stages of apoptosis increased (Figure 2C). Moreover, pretreatment with the pan-caspase inhibitor Z-VAD (10 μM) protected DU145 cells from apoptosis (Figure 2D–F), suggesting that genistein induces caspase-dependent apoptosis. However, in HPrEC cells, neither caspase-3/7 activity nor the cleavage of caspase-3 showed significant changes after genistein treatment, and the percentage of apoptotic cells remained unchanged, even with Z-VAD pretreatment. Flow cytometry revealed a sub-G_0_/G_1_ peak indicative of apoptosis in genistein-treated DU145 cells (Figure 3A). When treated with various genistein concentrations, the G_2_/M phase proportion in DU145 cells increased in a concentration-dependent manner to 5.16%, 10.80%, 11.15%, and 12.48%, respectively, with no significant difference observed in normal HPrEC cells. Additionally, Z-VAD pretreatment significantly reduced the proportion of cells with hypodiploid DNA but had little effect on the cell cycle (Figure 3B).

### 3.3. ROS, Mitochondrial Function, and ATP Play Critical Roles in Genistein-Induced Cytotoxicity

**Figure 4:**
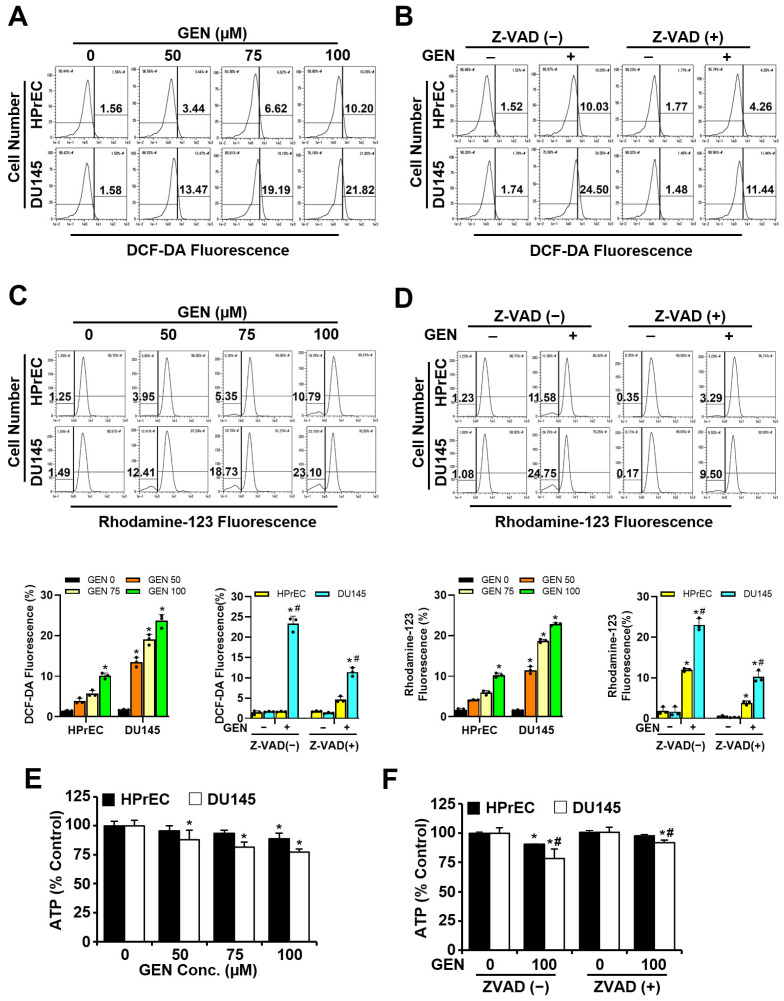
Effects of genistein and pan-caspase inhibitor on ROS and mitochondrial function in HPrEC and DU145 cells. Cells were treated with Z-VAD (10 μM) for 2 h before incubation with genistein (100 μM) for 48 h. (**A**,**B**) Cells were subsequently analyzed using flow cytometry after staining with propidium iodide (20 μg/mL). (**C**,**D**) Mitochondrial membrane potential was measured after staining the cells with rhodamine123. (**E**,**F**) Intracellular ATP levels. Representative results were obtained from one of three independent experiments. The quantitative data are presented as mean ± S.D. * *p* < 0.05 was compared to respective controls. # *p* < 0.05 was compared to Z-VAD (−) groups. GEN: genistein; HPrEC: human prostate epithelial cell line.

To determine if genistein increases ROS in prostate cancer cells, we conducted DCF-DA fluorescence assays. After treating HPrEC and DU145 cells with genistein at concentrations of 0, 50, 75, and 100 μM for 48 h, ROS levels were quantified using DCF-DA fluorescence. The results indicated that ROS levels in DU145 cells increased in a concentration-dependent manner by 13.47%, 19.19%, and 21.82%, respectively, relative to the control. Conversely, in HPrEC cells, ROS levels did not significantly change with increasing genistein concentrations (Figure 4A). When HPrEC and DU145 cells were treated with genistein (100 μM) and Z-VAD (10 μM) either singly or in combination to assess intracellular ROS levels, the ROS levels in DU145 cells rose to 24.5% in the genistein-only treatment group compared to the control. In the combined treatment group with genistein and Z-VAD, the increase was 11.44%, which was lower than that observed with genistein alone. ROS levels in HPrEC cells did not show significant change when treated with genistein and Z-VAD either alone or in combination (Figure 4B). Subsequently, we utilized rhodamine123 fluorescence to evaluate the impact of genistein on mitochondrial function in prostate cells. After treating HPrEC and DU145 cells with genistein for 48 h at the same concentrations, the percentage of mitochondrial membrane potential collapse in DU145 cells increased in a concentration-dependent manner to 12.41%, 18.73%, and 23.10%, respectively, compared to the control. In HPrEC cells, the percentage of mitochondrial membrane potential collapse did not significantly change with increasing genistein concentrations (Figure 4C). Treatment of HPrEC and DU145 cells with genistein (100 μM) and Z-VAD (10 μM) singly or in combination resulted in an increased percentage of mitochondrial membrane potential collapse in DU145 cells up to 24.75% in the genistein-only treatment compared to the control, but only up to 9.5% in the combined treatment, which was less than the genistein-only treatment. The percentage indicating mitochondrial membrane potential collapse in HPrEC cells was not significant with either treatment (Figure 4D). We also conducted experiments to ascertain the effect of genistein on intracellular ATP levels. When HPrEC and DU145 cells were treated with genistein at concentrations of 0, 50, 75, and 100 μM for 48 h, the intracellular ATP levels did not significantly differ in HPrEC cells but decreased in DU145 cells as the genistein concentration increased compared to the control (Figure 4E). When HPrEC and DU145 cells were treated with genistein (100 μM) and Z-VAD (10 μM) either singly or in combination, the intracellular ATP level in DU145 cells treated with genistein alone decreased compared to the control but not significantly when genistein and Z-VAD were combined. Treatment of HPrEC cells with genistein and Z-VAD, either singly or in combination, showed no significant effect (Figure 4F). It was found that genistein increased ROS and decreased mitochondrial function in DU145 cells, leading to a decrease in intracellular ATP.

### 3.4. Genistein Promotes De Novo Synthesis, Leading to the Upregulation of p53 Expression in Prostate Cancer Cells

**Figure 5:**
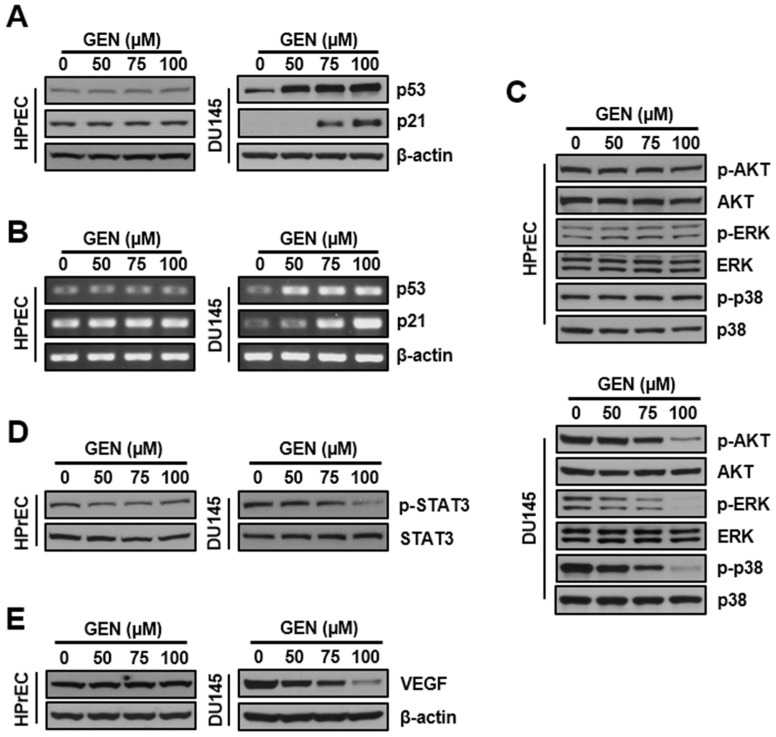
Effects of genistein on the p53, p21, AKT, ERK, MAPK, STAT3 pathways, and VEGF protein levels in HPrEC and DU145 cells. Cells were treated with increasing concentrations (0–100 μM) of genistein for 48 h. (**A**–**E**) The cell lysates were subjected to Western blot analysis targeting proteins such as p53, p21, p-AKT, AKT, p-ERK, ERK, p-p38, p38, p-STAT3, STAT3, and VEGF. GEN: genistein; HPrEC: human prostate epithelial cell line.

Genistein induces p53 upregulation at both the protein and mRNA levels. To identify the effector molecules responsible for genistein-induced apoptosis, we analyzed p53 expression following genistein treatment. As shown in Figure 5A, genistein treatment increased p53 protein expression in DU145 cells in a dose-dependent manner. In contrast, p53 levels in HPrEC cells remained unchanged after genistein treatment. Having demonstrated genistein’s preferential p53 upregulation in DU145 cells, we explored the mechanisms underlying this effect. With increasing genistein concentration, transcription levels of target genes such as p53 and p21 also increased following the treatment (Figure 5B).

### 3.5. Regulatory Expression of Proteins Related to MAPK and STAT3 Pathways in Prostate Cancer DU145 Cells Treated with Genistein

Western blotting was conducted to determine whether genistein regulates the expression of oncogenic signaling proteins, including the MAPK pathway and STAT3 pathways in HPrEC and DU145 cells. Treatment of cells with genistein (0, 50, 75, and 100 μM) for 48 h revealed that the expression of p-ERK, p-p38, and p-AKT proteins, which are involved in cell proliferation and survival, was concentration-dependently decreased in DU145 cells. However, no apparent changes were observed in the expression of p-ERK, p-p38, and p-AKT proteins in HPrEC cells (Figure 5C). In addition, the expression of p-STAT3 and its downstream transcription target VEGF, which are involved in cancer cell growth, angiogenesis, and metastasis, were also decreased in a dose-dependent manner in DU145 cells but did not affect the expression of these proteins in HPrEC cells (Figure 5D,E).

## 4. Discussion

Prostate cancer ranks high among the causes of cancer deaths in men, and it is the second most common male cancer in Korea and globally [25]. Continuous development of anticancer drugs aims to treat prostate cancer; however, numerous studies have documented drug resistance and adverse effects. Significantly, the quality of life deteriorates in cancer patients undergoing chemotherapy due to these side effects. Consequently, research is ongoing to develop natural anticancer agents that exhibit fewer side effects and resistance than existing treatments. Over the past several decades, soy isoflavones, including genistein, daidzein, and equol, have been studied for their potential anticancer activities, in addition to their anti-inflammatory and antioxidant properties [26,27]. Their biological effects, including apoptosis, cell proliferation, migration/invasion, angiogenesis, and metastasis in prostate cancer cells, vary somewhat depending on the degree of efficacy and targets of action, but are achieved through interactions with various cellular signaling pathways.

Recent pharmacological studies have demonstrated that genistein, a flavonoid component found in soybeans, possesses anti-inflammatory, antiviral, anti-tumor, and antioxidant properties through its free radical scavenging ability [13,28]. This study was conducted to investigate the effects of genistein, an isoflavone extracted from soybeans, on prostate cancer cell lines using the DU145 prostate cancer cell line. The efficacy of genistein against the prostate cancer DU145 cell was confirmed by analyzing cell viability, protein expression, and cell death, and the basic mechanism of sensitivity to genistein was identified and four major factors were defined. First, genistein treatment caused cytotoxicity and cell cycle arrest, resulting in decreased cell viability. Second, genistein induced changes in apoptosis-related proteins, including an increase in the cleaved forms of Bax, procaspase-3, and PARP and a decrease in Bcl-2. Third, genistein inhibited the activation of these pathways by significantly reducing the phosphorylated forms of AKT, ERK, p38, and STAT3. Fourth, the effects of genistein treatment were underscored by the decrease in VEGF protein levels associated with metastasis

Previous studies have demonstrated that genistein possesses a broad spectrum of biological activities, including antiproliferative effects against various cancer cells [29,30]. Due to its multi-target mechanism and minimal systemic toxicity, genistein is considered a chemosensitizer that can help overcome chemotherapy resistance in tumor cells. In this study, we observed that genistein preferentially inhibited the growth of DU145 cells and activated apoptosis at given concentration (50–100 μM), while it had little toxic effect on normal HPrEC cells. The decrease in cell numbers in the genistein-treated group compared to the control group may be attributed to both cell death and inhibition of cell proliferation. DAPI analysis and cleavage analysis of caspase and PARP are recognized as markers of apoptosis, and flow cytometry is commonly used to detect and quantify apoptotic cells [31]. In DU145 cells, treatment with genistein for 48 h led to chromatin condensation and nuclear fragmentation as confirmed by DAPI analysis, and apoptosis-associated proteolytic cleavage of Bax, caspases-3, and PARP was detected by Western blotting. Here, enhanced cleavage of 21 kDa Bax to p18 Bax by cathepsin-like proteases is known to be an upstream signal for caspase-dependent apoptosis, increasing the intrinsic cytotoxic properties of Bax and accelerating its apoptotic function in mitochondria [32,33,34]. Increased sub-G_0_/G_1_ peak in flow cytometric analysis indicates that genistein, a soy-derived isoflavone, effectively inhibited cell growth and induced apoptosis in prostate cancer cells. Half of cancers are associated with inactivated p53, a critical sensor of genotoxic stress that responds to cellular stressors, including cell death, DNA damage, angiogenesis, hypoxia, and activated oncogenes [35]. Our findings, which showed that genistein concentration-dependently increased cytotoxicity against DU145 cells, upregulated the mRNA and protein of p53 and its transcriptional target p21, and downregulated Bcl-2 expression, suggest that a p53-dependent pathway may be activated. Furthermore, p53 initiates an irreversible apoptotic program by activating the Bax gene [36], a key component of the Bcl-2 family, which interacts with Bcl-2 and promotes the production of apoptotic mediators such as caspase-3, thereby inhibiting cell cycle arrest at the G_2_/M phase. Further comparative studies in p53 null prostate cancer cell lines and additional analysis of more p53 transcriptional target genes are needed to confirm the p53-dependent effect of genistein on apoptosis.

Genistein is recognized for inhibiting VEGF expression and angiogenesis in tumors [37]. Overexpression of VEGF and hyperactivation of STAT3 are common in human tumors, and activation of STAT3 has been shown to upregulate VEGF expression and promote angiogenesis [38,39]. VEGF expression is associated with various signal transduction pathways, including transcription factor STAT3 and AKT or ERK pathways [40,41]. To elucidate the mechanism of genistein-induced angiogenesis in DU145 cells, we observed the activation of STAT3, AKT, and ERK signaling. Our findings indicated that genistein treatment reduced the phosphorylation levels of AKT, ERK, p38, and STAT3 in DU145 cells. STAT3 is an oncogenic transcription factor, constitutively activated in many human cancers, including prostate cancer, and activated by phosphorylation at Tyr^705^ in response to various signals such as cytokines and growth factors [42,43]. Phosphorylated STAT3 translocates to the nucleus and regulates the transcription and expression of a wide range of genes, including VEGF, Bcl-2, Mcl-1, survivin, D-cyclin, and the transcription factor E2F-1, thereby promoting tumor cell survival, proliferation, angiogenesis, tissue invasion, and evasion of apoptosis [44]. A complex signaling network involving cross-talk and interaction with other cellular pathways important for malignant progression, including the PI3K/AKT/mTOR, MEK/ERK, and p38 MAPK pathways, influences the STAT3 signaling pathway and its outcomes [45,46]. Therefore, various pharmacological approaches targeting STAT3 hyperactivation have been considered as potential strategies for the development of anticancer agents [47].

**Figure 6:**
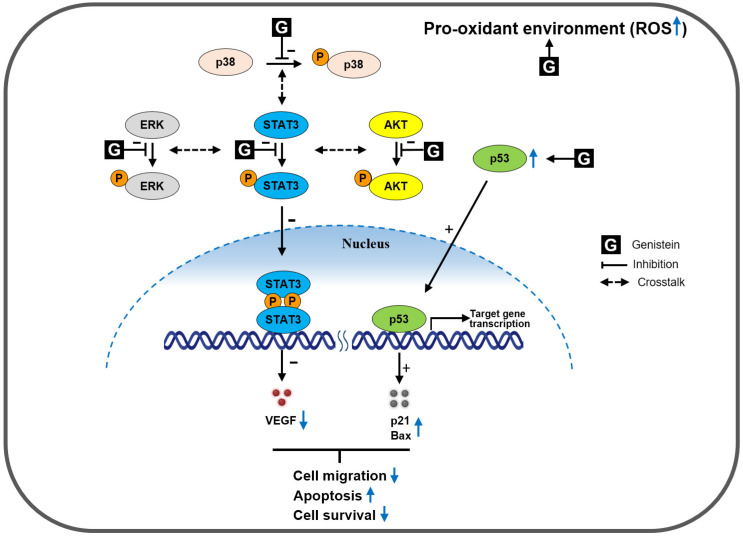
Schematic representation of the proposed mechanism for genistein-induced cytotoxicity in prostate cancer cells. Genistein inhibits the growth of prostate cancer DU145 cells and induces apoptosis by simultaneously inhibiting the activation of STAT3 and other closely related oncogenic kinases, such as AKT, ERK, and p38, and reducing VEGF expression.

In conclusion, this study provides a molecular basis for a novel role of genistein in simultaneously inhibiting the activation of STAT3 and other closely related oncogenic kinases, such as AKT, ERK, and p38, in prostate cancer DU145 cells (Figure 6). Genistein derived from soybeans may be considered a valuable therapeutic candidate for prostate cancer, but further experimental validation is needed to gain a comprehensive mechanistic understanding of genistein, particularly focusing on cross-talks between STAT3 and other signaling pathways in cell growth inhibition and apoptosis induction. In addition, for practical application of genistein in the prevention and treatment of prostate cancer, studies on the dose at which genistein reaches tissues should be conducted in advance.

## Data Availability

All data produced or assessed during this study are included in this article.

## References

[1] Shafiee G., Saidijam M., Tayebinia H., Khodadadi I. (2022). Beneficial effects of genistein in suppression of proliferation, inhibition of metastasis, and induction of apoptosis in PC3 prostate cancer cells. Arch. Physiol. Biochem..

[2] Wasim S., Lee S.Y., Kim J. (2022). Complexities of Prostate Cancer. Int. J. Mol. Sci..

[3] Sekhoacha M., Riet K., Motloung P., GoMenku L., Adegoke A., Mashele S. (2022). Prostate cancer review: Genetics, diagnosis, treatment options, and alternative approaches. Molecules.

[4] Teo M.Y., Rathkopf D.E., Kantoff P. (2019). Treatment of advanced prostate cancer. Annu. Rev. Med..

[5] Ahdoot M., Lebastchi A.H., Turkbey B., Wood B., Pinto P.A. (2019). Contemporary treatments in prostate cancer focal therapy. Curr. Opin. Oncol..

[6] Marchetti C. (2022). Calcium signaling in prostate cancer cells of increasing malignancy. Biomol. Concepts.

[7] Namekawa T., Ikeda K., Horie-Inoue K., Inoue S. (2019). Application of prostate cancer models for preclinical study: Advantages and limitations of cell lines, patient-derived xenografts, and three-dimensional culture of patient-derived cells. Cells.

[8] Swetha G M., Keerthana C.K., Rayginia T.P., Anto R.J. (2022). Cancer chemoprevention: A strategic approach using phytochemicals. Front. Pharmacol..

[9] Naeem A., Hu P., Yang M., Zhang J., Liu Y., Zhu W., Zheng Q. (2022). Natural products as anticancer agents: Current status and future perspectives. Molecules.

[10] Ahmed M.B., Islam S.U., Alghamdi A.A.A., Kamran M., Ahsan H., Lee Y.S. (2022). Phytochemicals as chemo-preventive agents and signaling molecule modulators: Current role in cancer therapeutics and inflammation. Int. J. Mol. Sci..

[11] Hao Q., Wu Y., Vadgama J.V., Wang P. (2022). Phytochemicals in inhibition of prostate cancer: Evidence from molecular mechanisms studies. Biomolecules.

[12] Dixon R.A., Ferreira D. (2002). Genistein. Phytochemistry.

[13] Khan F.B., Yahya P.S., Jamous F., Ali S.A., Abdullah, Uddin S., Zia Q., Jena M.K., Khan M., Owais M. (2022). Multifaceted pharmacological potentials of curcumin, genistein, and tanshinone IIA through proteomic approaches: An in-depth review. Cancers.

[14] Sharifi-Rad J., Quispe C., Imran M., Rauf A., Nadeem M., Gondal T.A., Ahmad B., Atif M., Mubarak M.S., Sytar O. (2021). Genistein: An integrative overview of its mode of action, pharmacological properties, and health benefits. Oxid. Med. Cell. Longev..

[15] Gupta N., Gupta S., Kumar M., Guarve K., Dhanawat M., Sharma V. (2023). Therapeutic potential of genistein and its derivatives as a target for anticancer agents. Chem. Select.

[16] Elmore S. (2007). Apoptosis: A review of programmed cell death. Toxicol. Pathol..

[17] Kumari S., Sharma V., Tiwari R., Maurya J.P., Subudhi B.B., Senapati D. (2022). Therapeutic potential of p53 reactivation in prostate cancer: Strategies and opportunities. Eur. J. Pharmacol..

[18] Li J., Li Y., Chen L., Yu B., Xue Y., Guo R., Su J., Liu Y., Sun L. (2020). p53/PGC-1α-mediated mitochondrial dysfunction promotes PC3 prostate cancer cell apoptosis. Mol. Med. Rep..

[19] Hassin O., Oren M. (2023). Drugging p53 in cancer: One protein, many targets. Nat. Rev. Drug Discov..

[20] Chi S.W. (2014). Structural insights into the transcription-independent apoptotic pathway of p53. BMB Rep..

[21] Miklossy G., Hilliard T.S., Turkson J. (2013). Therapeutic modulators of STAT signalling for human diseases. Nat. Rev. Drug Discov..

[22] Corvinus F.M., Orth C., Moriggl R., Tsareva S.A., Wagner S., Pfitzner E.B., Baus D., Kaufmann R., Huber L.A., Zatloukal K. (2005). Persistent STAT3 activation in colon cancer is associated with enhanced cell proliferation and tumor growth. Neoplasia.

[23] Siveen K.S., Sikka S., Surana R., Dai X., Zhang J., Kumar A.P., Tan B.K.H., Sethi G., Bishayee A. (2014). Targeting the STAT3 signaling pathway in cancer Role of synthetic and natural inhibitors. Biochim. Biophys. Acta.

[24] Wendt M.K., Balanis N., Carlin C.R., Schiemann W.P. (2014). STAT3 and epithelial–mesenchymal transitions in carcinomas. JAK-STAT.

[25] Park S.K., Sakoda L.C., Kang D.H., Chokkalingam A.P., Lee E.S., Shin H.R., Ahn Y.O., Shin M.H., Lee C.W., Lee D.H. (2006). Rising prostate cancer rates in South Korea. Prostate.

[26] Mahmoud A.M., Yang W., Bosland M.C. (2014). Soy isoflavones and prostate cancer: A review of molecular mechanisms. J. Steroid Biochem. Mol. Biol..

[27] Křížová L., Dadáková K., Kašparovská J., Kašparovský T. (2019). Isoflavones. Molecules.

[28] Atanasov A.G., Zotchev S.B., Dirsch V.M., Supuran C.T. (2021). Natural products in drug discovery: Advances and opportunities. Nat. Rev. Drug Discov..

[29] Ye R., Bodero A., Zhou B.B., Khanna K.K., Lavin M.F., Lees-Miller S.P. (2001). The plant isoflavenoid genistein activates p53 and Chk2 in an ATM-dependent manner. J. Biol. Chem..

[30] Lian F., Li Y., Bhuiyan M., Sarkar F.H. (1999). p53-independent apoptosis induced by genistein in lung cancer cells. Nutr. Cancer.

[31] Steensma D.P., Timm M., Witzig T.E. (2003). Flow cytometric methods for detection and quantification of apoptosis. Methods Mol. Med..

[32] Tamura H., Ohtsuru A., Kamohara Y., Fujioka H., Yanaga K., Kanematsu T., Yamashita S. (2003). Bax cleavage implicates caspase-dependent H2O2-induced apoptosis of hepatocytes. Int. J. Mol. Med..

[33] Cao X., Deng X., May W.S. (2003). Cleavage of Bax to p18 Bax accelerates stress-induced apoptosis, and a cathepsin-like protease may rapidly degrade p18 Bax. Blood.

[34] Wood D.E., Newcomb E.W. (2000). Cleavage of Bax enhances its cell death function. Exp. Cell Res..

[35] Brady C.A., Attardi L.D. (2010). p53 at a glance. J. Cell Sci..

[36] Burns T.F., Fei P., Scata K.A., Dicker D.T., El-Deiry W.S. (2003). Silencing of the novel p53 target gene Snk/Plk2 leads to mitotic catastrophe in paclitaxel (taxol)-exposed cells. Mol. Cell. Biol..

[37] Cheng W.X., Huang H., Chen J.H., Zhang T.T., Zhu G.Y., Zheng Z.T., Lin J.T., Hu Y.P., Zhang Y., Bai X.L. (2020). Genistein inhibits angiogenesis developed during rheumatoid arthritisthrough the IL-6/JAK2/STAT3/VEGF signalling pathway. J. Orthop. Transl..

[38] Loeffler S., Fayard B., Weis J., Weissenberger J. (2005). Interleukin-6 induces transcriptional activation of vascular endothelial growth factor (VEGF) in astrocytes in vivo and regulates VEGF promoter activity in glioblastoma cells via direct interaction between STAT3 and Sp1. Int. J. Cancer.

[39] Yang L., Wang L., Lin H.K., Kan P.Y., Xie S., Tsai M.Y., Wang P.H., Chen Y.T., Chang C. (2003). Interleukin-6 differentially regulates androgen receptor transactivation via PI3K-Akt, STAT3, and MAPK, three distinct signal pathways in prostate cancer cells. Biochem. Biophys. Res. Commun..

[40] Wang Y., Crisostomo P.R., Wang M., Markel T.A., Novotny N.M., Meldrum D.R. (2008). TGF-alpha increases human mesenchymal stem cell-secreted VEGF by MEK- and PI3-K- but not JNK- or ERK-dependent mechanisms. Am. J. Physiol. Regul. Integr. Comp. Physiol..

[41] Wu X., Chen Z., Zeng W., Zhong Y., Liu Q., Wu J. (2015). Silencing of Eag1 gene inhibitsosteosarcoma proliferation and migration by targeting STAT3-VEGF pathway. BioMed Res. Int..

[42] Azare J., Leslie K., Al-Ahmadie H., Gerald W., Weinreb P.H., Violette S.M., Bromberg J. (2007). Constitutively activated Stat3 induces tumorigenesis and enhances cell motility of prostate epithelial cells through integrin beta 6. Mol. Cell. Biol..

[43] Chung S.S., Aroh C., Vadgama J.V. (2013). Constitutive activation of STAT3 signaling regulates hTERT and promotes stem cell-like traits in human breast cancer cells. PLoS ONE.

[44] Zhang Z., Mao H., Du X., Zhu J., Xu Y., Wang S., Xu X., Ji P., Yu Y., Cao B. (2016). Novel small molecule agent displays potent anti-myeloma activity by inhibiting the JAK2-STAT3 signaling pathway. Oncotarget.

[45] Ogunwobi O.O., Beales I.L. (2007). The anti-apoptotic and growth stimulatory actions of leptin in human colon cancer cells involves activation of JNK mitogen activated protein kinase, JAK2 and PI3 kinase/Akt. Int. J. Color. Dis..

[46] Wang X., Crowe P.J., Goldstein D., Yang J.L. (2012). STAT3 inhibition, a novel approach to enhancing targeted therapy in human cancers (review). Int. J. Oncol..

[47] Zarezadeh S.M., Sharafi A.M., Erabi G., Tabashiri A., Teymouri N., Mehrabi H., Golzan S.A., Faridzadeh A., Abdollahifar Z., Sami N. (2024). Natural STAT3 inhibitors for cancer treatment: A comprehensive literature review. Recent Pat. Anticancer Drug Discov..

